# Measuring Mechanism and Applications of Polymer-Based Flexible Sensors

**DOI:** 10.3390/s19061403

**Published:** 2019-03-21

**Authors:** Zewen Yang, Hong Xu, Yao Huang, Jingyao Sun, Daming Wu, Xiaolong Gao, Yajun Zhang

**Affiliations:** Mechanical and Electrical Engineering Institute, Beijing University of Chemical Technology, 15 Beisanhuan Dong Lu, Chaoyang District, Beijing 100029, China; 2016200610@mail.buct.edu.cn (Z.Y.); huangyao@mail.buct.edu.cn (Y.H.); sunjingyao5566@sina.com (J.S.); wudm@mail.buct.edu.cn (D.W.); gaoxiaolong@mail.buct.edu.cn (X.G.); zhyj@mail.buct.edu.cn (Y.Z.)

**Keywords:** polymer-based flexible sensor, forced assembly, dynamic alternating load, same deformation characteristics

## Abstract

A new type of flexible sensor, which could maintain the deformation consistency and achieve the real-time detection of the variation in load of the measured object, was proposed in this work. According to the principle of forced assembly, PDMS was used as the substrate of sensitive components and electrodes, while carbon fiber was added as a conductive medium to prepare a polymer-based flexible sensor, which effectively overcame the deformation limitation and output instability of conventional flexible sensors due to different substrates of sensitive components and the electrode. Combined with the sensor structure and the forced assembly method, a theoretical analysis of its conductive measurement mechanism was carried out. Meanwhile, an experimental test device was designed to test and analyze the output characteristics of the flexible sensor under a static and dynamic alternating load. The results show that the flexible sensor exhibited linear output under the dynamic alternating load of 10 kN to 60 kN and frequency of 3 Hz. Peak and valley value had the same phase with the load extremes. The dynamic and static experiments show that the resistance output signal and the sensitivity was in the range of 310~624.15 Ω and 171–183 N/Ω respectively. However, due to the hysteresis of the elastic recovery of the polymer, the output repeatability of the flexible sensor under the dynamic alternating load was 5.03% and 0.78% lower than that of the static load, respectively. Combined with the static and dynamic experiments, it was verified that the polymer-based flexible sensor can maintain the same deformation characteristics with the measured object, and at the same time outputted a resistance signal with a certain mapping relationship with the applied load. The repeatability of the output signal under dynamic and static experiments was within ±7%, which can meet the measurement requirements of the fatigue life of the measured body during periodic load.

## 1. Introduction

With the development of information technology, electronic technology and polymer synthesis processing technology, as a new type of sensor, the polymer-based flexible sensors have been widely used in the detection of human motion states [1], physiological parameters detection on skin [2,3], environmental signal monitoring [4] and other complex situations that the shape of measured objects would change with variation forces, attributed to their superior flexibility, malleability, portability and wearability [5,6,7].

Polydimethylsiloxane (PDMS), Poly(ethylene terephthalate) (PET), polyimide (PI) and parylene, which has superior flexibility, malleability, were often used as the substrate for the flexible sensor. The electrodes were usually made of carbon nanotubes (CNTs), graphene (GN), carbon fiber (CF) and nano-metal materials as the main conductive medium, prepared by different molding methods [8,9,10]. JS et al. [11] from Seoul National University made a super thin flexible sensor with a thickness of about 1/6 of human skin using poly(vinylidene fluoride) (PVDF) as the substrate and zinc oxide (ZnO) as the electrode. The pressure detection sensitivity was about 10 Pa and the temperature detection was in the range of 20~120 °C. Baek et al. [12] from Korea University made oxygen ion treatment on the surface of PDMS film with 3 mm thick protrusion structure, and then bonded with PDMS film containing copper metal layer to make flexible sensor for electrocardiogram (ECG) signal detection. Ruan X et al. [13] from Xi’an University of Architecture and Technology, using CNTs as conductive filler and PDMS as the matrix material to make the flexible sensor, which was prepared by solution method, could realize real-time monitoring of pressure distribution and size. However, when the electrode with nano-metal material as the conductive medium met larger deformation, it would cause a break and cause the discontinuity of electrical property conduction, affecting the output characteristics of the sensor. In addition, electrodes prepared by composite materials with dissimilar polymer substrates often have inconsistent deformations under stress due to the difference in physical properties of the two materials. The range of force deformation of polymer-based flexible sensor would be affected according to these factors, which therefore caused a limitation when the sensor was working.

In this paper, PDMS was used as the substrate of the polymer-based flexible sensor, and the sensing elements and electrodes were assembled with carbon fiber as the conductive medium by a spatial confining forced network assembly method [14,15,16]. The sensing elements and the electrodes material, which were made of the same material, have the same telescopic matching property, which could improve the working range of deformation of the sensor and measured component under periodic load. Meanwhile, it could make the output signal have a great mapping relationship with the force within a certain range, and had stable repeatability and accuracy.

## 2. Structural Design and Measuring Mechanism of Polymer-Based Flexible Sensors

### 2.1. Structural Design of Polymer-Based Flexible Sensors

**Figure 1:**
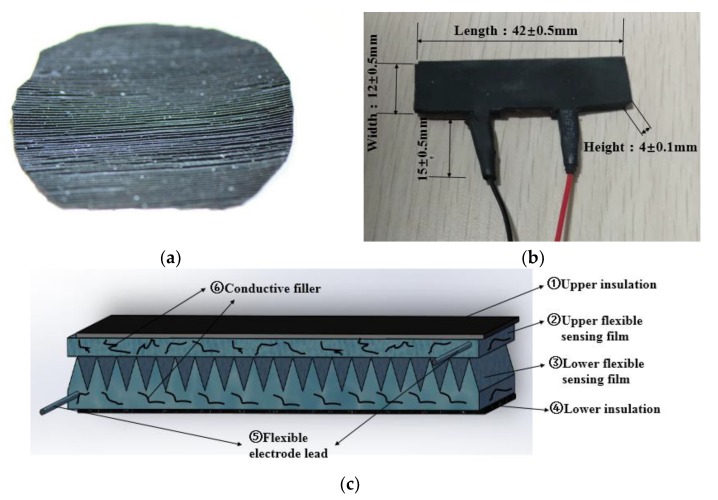
Polymer-based piezo-resistive flexible sensor. (**a**) Micro-structured film. (**b**) Sensor. (**c**) Theoretical diagram.

In the preparation process, after adding the relevant conductive medium (CF, CNTs) in proportion in A glue, we then sent the A glue to the internal mixer for thorough mixing. After evenly mixing with the B glue, the whole material was sent to a vacuum drying oven for a vacuuming operation. Finally, the material was evenly placed on the surface of the mold with a triangular microstructure. After the secondary compression molding process which included pressure holding and imprinting stages was carried out in a hot coining machine by a space-limited forced assembly method, a lower flexible sensing film having a microgroove structure was formed. The upper sensing film without the micro-groove structure and the electrode were prepared using the microstructure-free mold under the same process conditions [17,18,19,20]. Finally, a piezo-resistive sensor was synthesized by encapsulating the bond with the polymer insulating layer. The actual structure and theoretical diagram are shown in Figure 1b,c.

As shown in Figure 1c, the structure of the piezo-resistive flexible sensor was totally different from the conventional diffusion silicon piezo-resistive pressure sensor and the grid-shaped strain gauge. The flexible sensor was composed of an upper flexible sensing film ② and a lower flexible sensing film ③ made of the same process recipe, wherein the surface of the lower flexible sensing film ③ was formed into a microstructure groove. The positive and negative electrodes ⑤, ⑥ of the sensor were made up of the same materials with the sensing films ②, ③ and the same processing method. They were located at the two ends of the micro structured sensing films. Finally, the sensing film and the electrode were bonded together by the upper and lower insulating layers ①, ④, which were prepared with pure polymer to form a sensor.

### 2.2. Structure Design of Sensing Film

Before the static and dynamic experiments, the conductive properties of the sensing film were analyzed by controlling the thickness of the film and the microstructure array. The purpose is to obtain a conductive material with good comprehensive performance, which plays an important role in the preparation of flexible pressure sensors.

In the experimental aspect, the conductive properties of the sensing film with a thickness of 1 mm to 4 mm and the triangular and columnar arrays of the same height were analyzed. The film used for the measurement was cut into a circular shape with a diameter of 10 mm.

**Figure 2:**
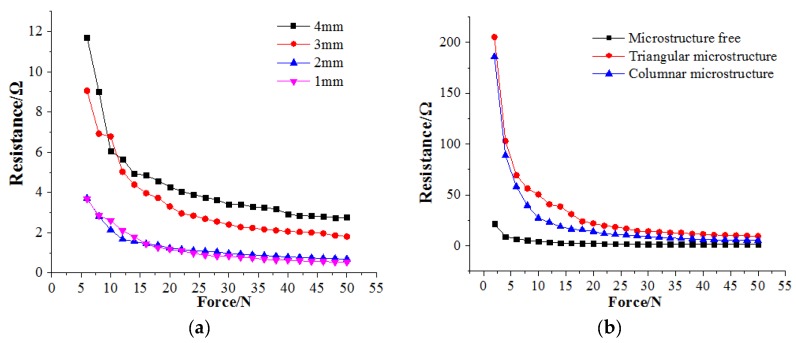
Piezo-resistive properties of different thickness and microstructure arrays. (**a**) Piezo-resistive properties of different thicknesses. (**b**) Piezo-resistive properties of different microstructures.

First, the effects of different thicknesses on the performance of the sensing film were analyzed. Through Figure 2a, it can be seen that the resistance changes with force under the thickness of 1mm and 2 mm were basically the same, the size above 2 mm is relatively thick, and may cause a sealing problem and micro structure damage in the sealing and work process. Combined with the analysis of advantages and disadvantages, 1 mm was selected as the thickness of the actual packaging application.

The height of the three microstructures in Figure 2b was 1.5 mm. It can be seen that the triangular microstructure had a relatively wide resistance change interval compared with the other two structures, and the change was stable at the same time. It had better resistance-force matching for practical applications.

In view of the above, the size of the sensor-sensing film herein was finally determined, as shown in Section 4.2.

### 2.3. Measuring Mechanism of Polymer-Based Flexible Sensors

The resistance output signal of the conventional grid-shaped strain gauge and the diffused silicon piezo-resistive pressure sensor would change under shape deformations caused by tensile deformation and vertical pressure. Resistance variation of these sensitive components was shown in Equation (1) [21].

(1) $$\frac{\Delta R}{R}=\frac{\Delta \rho }{\rho }+\frac{\Delta l}{l}-\frac{\Delta S}{S},$$

where *R* is the resistance of the conductor or the semiconductor, *ρ* is the resistivity of the conductor or the semiconductor; *l* is the length of the conductor, mm; *S* is the cross-sectional area of the conductor, mm^2^; *ΔR* is the change of the resistance under the external force, Δl, *ΔS* are the changes of the length and area of the conductive grid under the action of external force, and *Δρ* is the change of the conductivity of the semiconductor under the action of external force. The rate of change of the resistance of the diffused silicon pressure sensor and the strain gauge is caused by the first term and the second term of the Equation (1) respectively.

**Figure 3:**
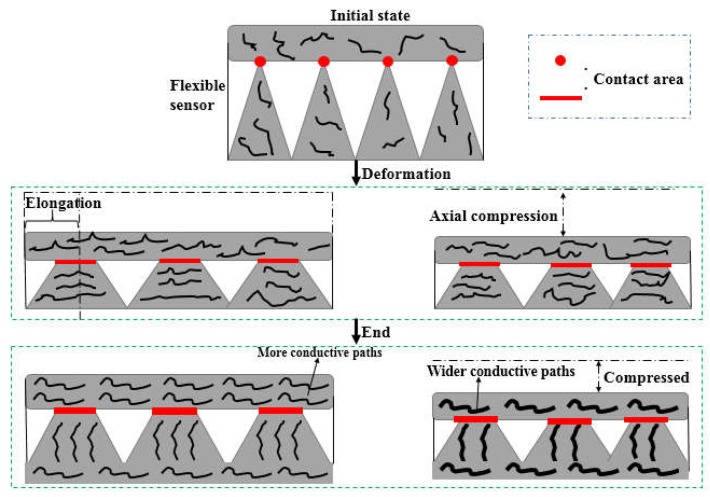
Schematic diagram of piezo-resistive properties of polymer-based flexible sensor.

During the polymer-based flexible sensor packaging process, a certain preload was applied to the conductive films by the upper and lower polymer insulating layers, so that the upper and lower conductive films of the sensor contacted each other to generate a conductive path in the initial state. The measurement mechanism was shown in Figure 3. During the loading process, the resistance changes with load, which caused a significant change in the contact area between the upper and lower conductive film of the sensor. Meanwhile, the conductive medium in the upper and lower conductive film produced a certain orientation, which allowed the polymer matrix and the conductive medium to be squeezed by space due to compression in the direction of the force. The polymer between the conductive particles was forced to “squeeze”, resulting in a decrease in the spacing between the conductive particles and a change in the structure of the conductive path between the conductive films. Meanwhile, the path of the conductive network was wider and more encrypted. Eventually it caused a change in the resistance of the sensing film.

**Figure 4:**
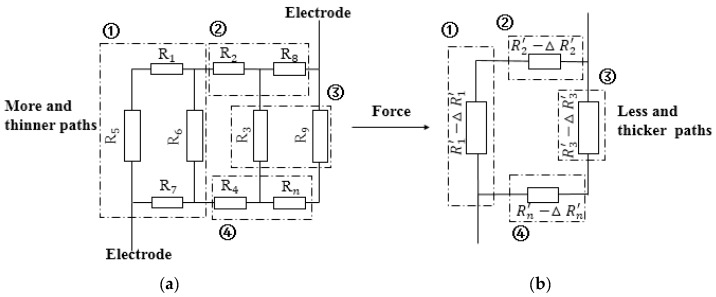
Equivalent circuit diagrams of resistance change of polymer-based flexible sensor. (**a**) Initial state. (**b**)Pressed state.

The resistance change process of the sensor in Figure 3 could be further elaborated by an equivalent circuit: the contact area of the upper and lower conductive films was increased due to the thickness reduction of the sensing film under external force, and the conductive particles were more likely to form a conductive path. At the same time, because the spacing of the conductive particles decreased, the number of open circuits decreased, the conductive paths merged with each other, and a plurality of distributed resistors in the initial state were combined into a resistor through series and parallel connection. The equivalent circuit schematic was illustrated in Figure 4.

In the initial state, resistance $R_{Initial}$ of the polymer-based flexible sensor is based on Equation (2). After the external load is applied, the equivalent circuit diagram of the internal conductive path is changed from Figure 4a,b. The $R_{Forced}$ force is calculated by Equation (3). The equivalent circuit diagram of the internal conductive path was changed from Figure 4a,b when the flexible sensor under external load. The $R_{1}^{\prime }-△R_{1}^{\prime }$ of the module ① in Figure 3b was a combination of $R_{1}↽,R_{5}↽,R_{6}{\;\mathrm{and}\;R}_{7}$ of the module 1 in Figure 4a. This corresponded with the combination of the conductive paths described above. The remaining modules were similar, and the resistance of the circuit was significantly reduced compared with the initial state after the load was applied.

(2) $$R_{Initial}=f(R_{1},R_{2},\cdot \cdot \cdot R_{n}),$$

(3) $$R_{Forced}=f({R^{\prime }}_{1}-\Delta {R^{\prime }}_{1},{R^{\prime }}_{2}-\Delta {R^{\prime }}_{2},{R^{\prime }}_{3}-\Delta {R^{\prime }}_{3},{R^{\prime }}_{4}-\Delta {R^{\prime }}_{4}),$$

where, $R_{Initial}$ is the initial resistance of the sensor; $\;R_{n}$ is the resistance of each path in initial state; $R_{n}^{\prime }$ is the resistance of the combined conduction paths; Δ$R_{n}^{\prime }$ is the resistance which decreases due to a decrease in the spacing of the conductive particles.

## 3. Signal Acquisition and Processing System of Flexible Sensors

**Figure 5:**
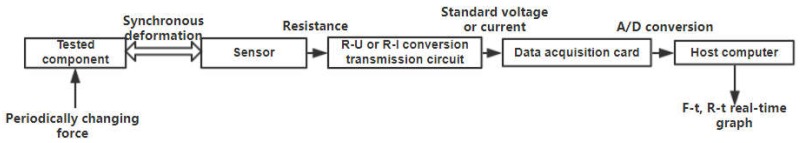
Polymer-based flexible sensor signal acquisition and processing flow chart.

The polymer-based flexible sensor signal acquisition and processing flow chart was shown in Figure 5.

The polymer-based flexible sensor was fixed on the side of the measured elastic element. with the external load, the sensor and the measured elastic component had a uniform deformation under the dynamic alternating load, and the output resistance signal had a mapping relationship. The resistance signal was converted to a standard voltage or current signal (0 to 10 V or 4 mA to 20 mA) by a resistor-voltage/current (RV/I) transmitter, and then converted to a digital value by a standard data acquisition card (A/D conversion). Finally, after being processed by the host computer software, the real-time curve of the pressure F of the sensor and the resistance R of the sensor with time T was obtained.

## 4. Experimental Study of Polymer-Based Flexible Sensors under Periodic Alternating Load

### 4.1. The Loaded State of the Measured Component

**Figure 6:**
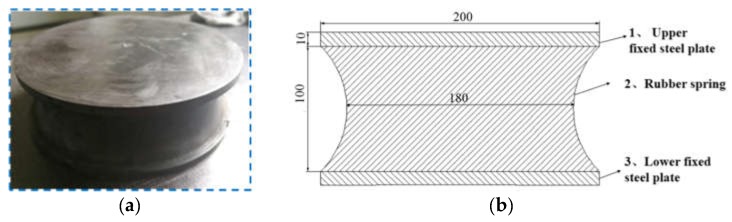
Shape and structure dimension of the measured component. (**a**) Outline. (**b**) Two-dimensional section view.

The measured component—dumbbell-shaped rubber high-elastic spring, was shown in Figure 6a: First standard rubber was the material and main filler was carbon black. The structure size was shown in Figure 6b: the upper and lower ends were supported by a 10 mm thick steel plate. When subjected to cyclic alternating load, the central part of the depressed area was subject to the greatest deformation and prone to fatigue damage.

**Figure 7:**
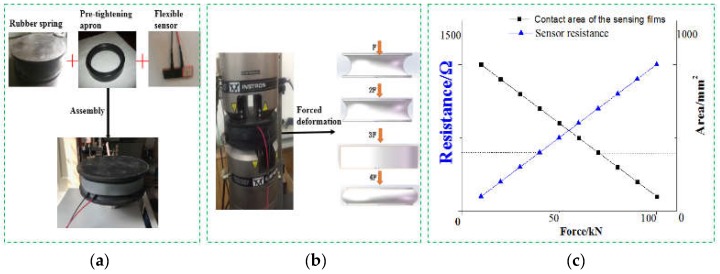
The installation and working diagram of sensor and spring. (**a**) Assembly. (**b**) Spring deformation diagram. (**c**) Piezo-resistive characteristics.

First of all, the force deformation performance analysis of the polymer-based flexible sensor and the measured spring is performed. As shown in Figure 7a, the sensor was fixed with a rubber band to the side of the measured elastic component and placed on the fatigue testing machine, as shown in Figure 7b. When the pressure on the tested component was increasing, the spring was compressed and the concave areas on both sides were gradually bulging outwards. The sensor fixed on it mainly produced two kinds of deformation: one was tensile deformation in the circumferential direction, the other one was compression deformation along the central direction.

Under the initial preload of the rubber band, the flexible sensor was in the maximum load state, and the resistance was at its minimum. When the load was applied, due to the continuous outer drum of the side of the spring, the generated surface internal stress along the axial center canceled a part of the initial preload applied to the shaft on the sensor, so that the resultant force acting on the sensor was reduced. The contact area of two sensing films was continuously reduced, causing the resistance to increase as shown in Figure 7c, and the shape of the sensor was restored to some extent. This matched the measurement mechanism described above.

### 4.2. Experimental Equipment

**Figure 8:**
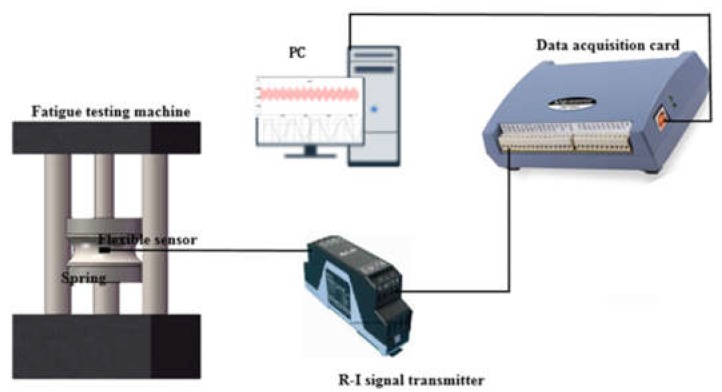
Overall diagram of the testing device.

A periodic alternating load was applied to the measured component through the fatigue testing machine. The signal conversion and transmission were realized through the transmitter and data acquisition card. A physical diagram of the test device and related components was shown in Figure 8.

(1) Fatigue testing machine

Model INSTRON-8801, loading range: 0~100 kN; Frequency range: 1~10 Hz; manufacturer: Instron (Shanghai) Test Equipment Trading Co., Ltd. (Shanghai, China);

(2) Polymer-based flexible piezo-resistive sensor

The substrate of sensing film was PDMS matrix filled with carbon fibers in a certain proportion. The upper film was a cubic structure with a size of 40 mm × 10 mm × 1 mm (length × width × height); The lower film was divided into two layers, where the lower layer was a cubic structure in the same size with the upper film and the upper layer was a micro-triangular groove array with a micro-groove size of 0.5 mm × 1.5 mm.

(3) Dumbbell-shaped rubber high-elastic spring component

The material was nitrile rubber with a shore hardness of 65~75 A. Elastic modulus 2.80 MPa. A definite ratio of carbon black was also filled.

(4) Preloading element—sealing rubber ring

The material was waterproof nitrile rubber with a thickness of 5 mm and an outer diameter of 150 mm.

(5) Signal transmitter

Model KL-N3511, input range 100–3000 Ω, output range 4~20 mA; manufacturer: Beijing Kunlun Coast Sensing Technology Co., Ltd.(Beijing, China);

(6) Data acquisition card:

Model USB-1608G, 16-bit conversion accuracy, standard current signal input, 4~20 mA, frequency 10 HZ; manufacturer: Shanghai Chengke Electromechanical Equipment Co., Ltd. (Shanghai, China);

(7) Host computer processing system

Hardware CPU: ATmega328, the PC software system was Arduino (based on the C language). Serial communication with the data acquisition card was achieved through USB connection.

Components (2), (3), and (4) were packaged as shown in Figure 7a in a simulation experiment. With the preload of the rubber ring, the sensor was located in the maximum stress state and the initial resistance is 289 Ω. The resistance fluctuation rate of change with time was less than ±3%. When the load working on the spring increased, the load state of the sensor was in the force release state, and the resistance gradually raised, as shown in Figure 7c.

### 4.3. Results and Analysis

In order to verify the stability and reliability of the flexible sensor under cyclic load, the static load test and the dynamic alternating load test were performed in the actual working simulation environment of 10 kN to 60 kN.

#### 4.3.1. Static Force Analysis

**Figure 9:**
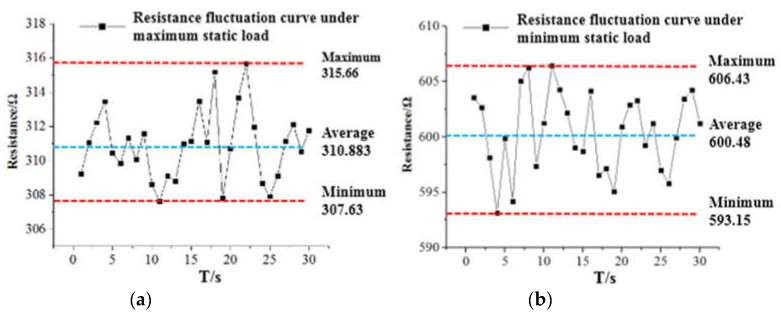
Resistance change diagrams for static extreme load. (**a**) Resistance change diagram at 10kN. (**b**) Resistance change diagram at 60kN.

Under the static experiment, the output characteristics of the sensor were tested at the extreme load of 10 kN and 60 kN respectively. The stability and repeatability were analyzed by multiple sampling. Figure 9a,b correspond to the output resistance changes of the sensor at 10 kN and 60 kN load respectively.

It can be seen from Figure 9a that the maximum fluctuation value of the resistance output corresponding to the minimum load was ±5 Ω, and the fluctuation error rate of change was ±1.61%. Similarly, the maximum fluctuation value of the resistance output corresponding to the maximum load was ±7 Ω, and the fluctuation error rate of change was ±1.17%, as shown in Figure 9b. Comparing the fluctuation error rate of change of ±3% in the initial state, the results show that the sensor was stable at the static load boundary.

**Figure 10:**
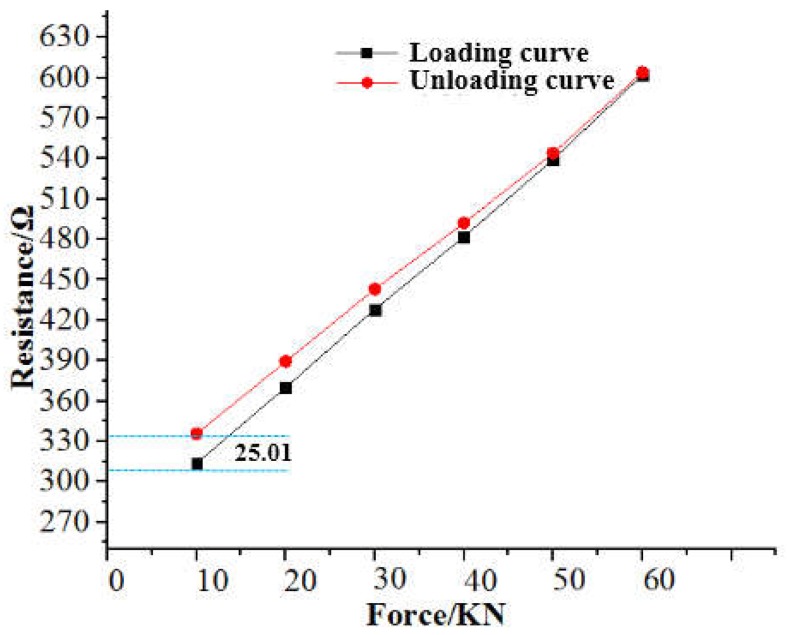
Polymer-based flexible sensor hysteresis characteristic curve.

The rapid change of the fixed frequency of the load in the dynamic test was distinguished, followed by the static loading and unloading experiment. The experiment started with the minimum load of 10 kN, and the load increased by 10 kN every 3 s, and then slowly loaded to the maximum load of 60 kN, and then unloaded to 10 kN in the same way, and recorded the data values of each point. Then, under the condition that the sensor was not taken out, 20 repeated tests were carried out, and the average value of 10 consecutive samples was randomly selected to calculate the average value. Loading-unloading resistance output characteristic curve was obtained as shown in Figure 10, and the analysis of the load-unloading hysteresis characteristic of the polymer-based flexible sensor was carried out.

As can be seen from the above figure, during the loading and unloading process, the output resistance of the sensor and the load of the device under test largely changed linearly. The resistance of the loading and unloading process was in the range of 310–602 Ω and 331–604 Ω respectively. Meanwhile, the sensitivity was 171 N/Ω and 183 N/Ω respectively. In addition, during the unloading process, as the applied load was gradually reduced, the backlash error gradually increased, and the return error reached a maximum value of 25.01 Ω at 10 kN, and the return error rate of change was 6.34%. In comparison, the two values were greater than the maximum return error of 3.56 Ω and return error rate of change of 0.51% at the 60 kN. Due to the change time of the loading frequency was less than the elastic recovery time of the flexible sensor, so that the error accuracy of the flexible sensor was reduced at the minimum load, and the fluctuation range of the wave at the valley was larger than that of the peak. This part will be more intuitive in the dynamic test.

#### 4.3.2. Dynamic Force Analysis

**Figure 11:**
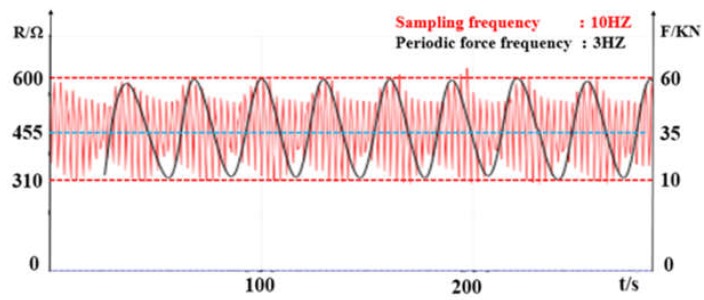
Waveform diagram of Sensor resistance signal and periodic force with time.

Unlike the static test condition, the fatigue testing machine performed a periodic dynamic load test with a frequency of 3 Hz and an alternating load of 10 kN to 60 kN sine wave, and obtained the load curve F-t and the corresponding sensor resistance output curve R-t as shown in Figure 11. The extreme value of the load in the Figure 11 corresponded to the extreme value of the output resistance, and the two phases were in phase (π/2 corresponded to the peak value, and 3π/2 corresponded to the trough value). The initial inner diameter of the central part of the spring was 180mm. Under the action of 10 kN to 60 kN, it increased to 206.87~235.57 mm, and the maximum elongation was 30.87%. According to the sensor output resistance signal, the tracking of the spring deformation signal can be well realized.

**Figure 12:**
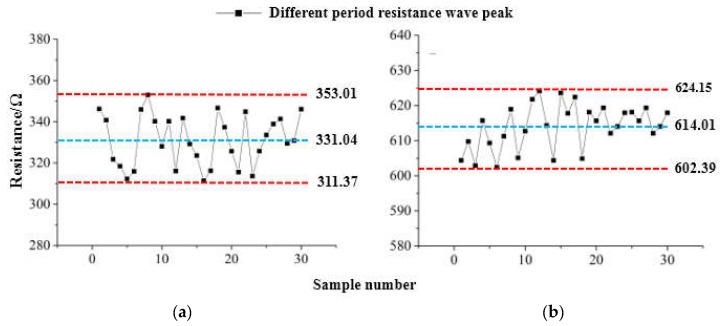
Schematic diagram of peak resistance in continuous cycle under dynamic load. (**a**) Resistance change diagram at 10kN. (**b**) Resistance change diagram at 60kN.

Under the dynamic alternating load, the average sample resistance at the extreme load of the sensor was in a range of 311.37~624.15 Ω. Minimum resistance was between 310 Ω and 331 Ω in static loading and unloading experimental data, and the maximum resistance exceeded 2% in the experimental static range. This was because the cumulative error caused by the polymer-based flexible sensor that produces maximum deformation under maximum alternating load but cannot be fully recovered. By sampling the resistance values corresponding to the minimum and maximum loads several times in succession, the output repeatability characteristics of the polymer flexible sensor under dynamic alternating load were analyzed. After the sampled values were digitally filtered, the resistance peak-to-valley curve of the flexible sensor under dynamic loading is obtained, as shown in Figure 12a,b.

It can be seen from Figure 12 that the maximum fluctuation value of the resistance output corresponding to the minimum load was ±22 Ω, the fluctuation error rate of change was ±6.64%, and the fluctuation range was about 4 times compared with the static load range. Similarly, the maximum fluctuation value of the resistance output corresponding to the maximum load was ±1 2Ω, the fluctuation error rate of change was ±1.95%, and the fluctuation range was less than 2 times compared with the same static load. It shows that under the cyclical alternating load, the output characteristics at the maximum load were much better than the output characteristics at the minimum load. This was mainly determined by the soft elastic recovery of the polymer flexible sensor substrate, and the experimental results were consistent with the experimental static conclusions. Under dynamic alternating load, when the pressure was gradually increased from 10 kN to 60 kN according to the sine law, the polymer-based flexible sensor reached the maximum with the deformation of the measured component. When the pressure was gradually reduced from 60 kN to 10 kN, the polymer-based flexible sensor was restored to the prototype with the deformation of the device under test. The accuracy within the cycle was still within ±7% of the project requirements.

## 5. Conclusions

Based on the above theoretical analysis and experimental investigation, it was verified that the polymer-based flexible sensor can maintain the synchronous deformation with the measured component when the load was applied, and the output resistance had a phase-matching relationship with the periodic alternating load. Under the load of 3 Hz and 10 kN to 60 kN, the sensor shows a linear variation between resistance and load. The resistance and sensitivity of dynamic and static load was in the range of 310~624.15 Ω and 171–183 N/Ω respectively, while the accuracy fluctuation range was within ±7%. In this work, the sensor based on the forced assembly method, which has certain measurement accuracy and repeatability, can meet the stress test of actual working conditions, and broke the deformation limitation of the elastic component for the traditional mechanical sensor. However, the flexible sensor had a certain zero drift due to the process error during packaging. Furthermore, due to the influence of the material resilience, the backlash error, which was difficult to eliminate, was generated at the extreme load and was more obvious at the minimum value, which gradually affected the repeatability of the test process. In addition, in the actual application process, it was necessary to consider the influence and compensation of the environmental factors such as the temperature on signal conversion and acquisition processing.
